# Characterization of Commercially Available Vaginal Lubricants: A Safety Perspective

**DOI:** 10.3390/pharmaceutics6030530

**Published:** 2014-09-22

**Authors:** Ana Raquel Cunha, Rita M. Machado, Ana Palmeira-de-Oliveira, José Martinez-de-Oliveira, José das Neves, Rita Palmeira-de-Oliveira

**Affiliations:** 1 CICS-UBI: Health Sciences Research Center, Faculty of Health Sciences, University of Beira Interior, Av. Infante D. Henrique, 6200-506 Covilhã, Portugal; E-Mails: ana_raquel222@hotmail.com (A.R.C.); rita.s.m.machado@gmail.com (R.M.M.); apo@fcsaude.ubi.pt (A.P.-d.-O.); jmo@fcsaude.ubi.pt (J.M.-d.-O.); 2 Labfit–Health Products Research and Development (HPRD), Lda, 6200-506 Covilhã, Portugal; 3 Child and Women Health Department, Centro Hospitalar Cova da Beira, 6200-251 Covilhã, Portugal; 4 INEB–Instituto de Engenharia Biomédica, University of Porto, 4150-180 Porto, Portugal; 5 CESPU, Instituto de Investigação e Formação Avançada em Ciências e Tecnologias da Saúde, 4585-116 Gandra PRD, Portugal; 6 Pharmacy Department, Centro Hospitalar Cova da Beira, 6200-251 Covilhã, Portugal

**Keywords:** vaginal drug delivery, microbicides, buffer capacity, osmolality, cytotoxicity

## Abstract

Vaginal lubricants are widely used by women to help solve intercourse difficulties or as enhancers, but recent reports raise questions about their safety. Twelve commercially available gel products were tested for pH value, pH buffering capacity, osmolality and cytotoxicity relevant to vaginal delivery. Obtained data were analyzed in light of the recent Advisory Note by the World Health Organization (WHO) for personal lubricants to be concomitantly used with condoms. Results showed that most products do not comply with pH and osmolality recommended standards, thus posing a potential hazard. Four products presented values of osmolality around three-times higher than the maximum acceptable limit of 1200 mOsm/kg. *In vitro* cell testing further identified substantial cytotoxicity even at 1:100 dilutions for three products, contrasting with no significant effect of up to at least a 1:5 dilution of a Universal Placebo gel. However, no direct correlation between these last results and pH or osmolality was found, thus suggesting that the individual toxicity of specific formulation components plays an important role in the outcome of a particular product. Although further assessment is required, these results highlight potential safety issues related to the formulation of commercially available vaginal lubricants.

## 1. Introduction

The importance of the vaginal route of drug delivery has been well established, both for local and systemic medical conditions [1,2]. Alongside therapeutic interest, different over-the-counter (OTC) products are routinely used as spermicides (*i.e.*, formulations containing spermicidal agents), or moisturizers, with cleansing or cosmetic purposes, or simply to assist sexual intercourse. Lubricants in particular are widely available and frequently used by women in order to allow the minimizing of dyspareunia or to enhance sexual pleasure [3]. These products are often available as water-based gels due to the interesting technological properties (e.g., easiness to produce and scale-up ability, versatile mechanical and rheological properties, affordability), bioadhesive properties, general condom compatibility, high user acceptability and the usually favorable safety profile of this semi-solid dosage form [4,5,6,7,8]. Other lubricant products based on different pharmaceutical systems are also available, but may present various disadvantages. For example, oil-based products are incompatible with latex condoms [9], while those including silicone are usually more expensive. Lubricants typically incorporate ingredients with GRAS status (generally recognized as safe substances, under 21 CFR part 182) or that are otherwise identified as non-toxic at recommended concentrations.

The U.S. Food and Drug Administration (FDA) and the European Medicine Agency (EMA), as well as most of the other regulatory bodies around the world, traditionally list lubricants as medical devices and relieve these products from extensive pre-clinical and clinical testing as otherwise required for drug products [10,11]. While there is a stringent lack of data on the safety of vaginal OTC lubricants, different *in vitro* and *in vivo* animal studies indicate that water-based lubricants may induce changes to the vaginal environment and mucosa that can lead to toxic effects and, eventually, enhancement of the transmission of sexually transmitted pathogens, such as HIV [12,13,14,15,16]. The World Health Organization (WHO), in collaboration with the United Nations Population Fund (UNFPA) and Family Health International (FHI360), recently recognized these risks and issued an “Advisory Note” on the technical requirements of lubricants, namely when used in addition to condoms [17]. Osmolality in particular has been highlighted, and specific recommendations have been proposed: values of 380 mOsm/kg or lower are desirable, but values as high as 1200 mOsm/kg have been considered acceptable on an interim basis. Further, other properties should be considered, namely intrinsic ingredient toxicity and pH. In this last case, deviations from the normal vaginal pH in the healthy adult (3.5–4.5 [2]) are considered as potentially deleterious.

In this study, a set of commercial lubricant gel products available on the international market was selected and evaluated *in vitro* for key features known to be relevant to the safety of vaginal products, namely pH and buffer capacity, osmolality and cytotoxicity. Results were discussed in view of the WHO recommendations [17] and data available in the literature.

## 2. Experimental Section

### 2.1. Materials

Hydroxyethylcellulose (HEC; Natrosol 250 HX) was kindly provided by Ashland Inc. (Covington, KY, USA). Sorbic acid and nonoxynol-9 (N-9; Tergitol type NP-9) were purchased from Sigma–Aldrich (Steinheim, Germany), and sodium chloride was acquired from Merck (Darmstadt, Germany). All other chemicals and reagents were of analytic grade or equivalent.

### 2.2. Tested Products

Twelve different gel-based lubricant products that are commercially available in various countries throughout Europe were included in this study, namely Fillergyn^®^ gel (BSDpharma, Lodi, Italy), Geliofil^®^ Classic gel (Laboratoires Effik, Meudon-la-Forêt, France), GelSea^®^ gel (LDPSA, Paris, France), Ginix^®^ gel (ISUS, Lisbon, Portugal), Ginix^®^ Plus gel (ISUS), Hyalo Gyn^®^ gel (Fidia Farmaceutici, Abano Terme, Italy), K-Y^®^ Jelly (Johnson & Johnson, Issy les Moulineaux, France), Phyto Soya^®^ gel (Arkopharma Laboratoires Pharmaceutiques, Carros, France), RepHresh^®^ gel (Lil’ Drug Store Products, Cedar Rapids, IA, USA), Replens^®^ gel (Lil’ Drug Store Products), Velastisa^®^ Intim VG moisturizer gel cream (Isdin, Barcelona, Spain) and Vidermina^®^ gel (Istituto Ganassini, Milano, Italy). Additional information on tested products is provided in the Table S1. For comparison purposes, a gel corresponding to the Universal Placebo [18,19] was also included in the subsequently described studies. The gel was prepared according to a previously described formula [18] by dissolving HEC (2.7 g) in water (96.3 g) containing sodium chloride (0.85 g) and sorbic acid (0.1 g). The final pH was adjusted to 4.4 by adding sodium hydroxide, and the gel was stored at 2–8 °C.

### 2.3. pH and Buffering Capacity Measurements

The pH of lubricants was measured at room temperature by directly immersing a pH electrode (Inlab^®^ Viscous, S220 SevenCompact™ pH meter, Mettler Toledo, Leicester, UK) into the samples. The buffering capacity of products was assessed by acid-base titration at room temperature, as previously described by Garg *et al.* [20]. Briefly, 1 M sodium hydroxide was added at 20 μL increments to 1 g of sample previously diluted in 10 mL of normal saline and under stirring. The pH was recorded after each addition and upon stabilization, until reaching a maximum pH value of 10. Obtained titration curves were used for determining the relevant buffering capacity, defined previously as the amount of sodium hydroxide required to reach a pH value of 5 [20]. Values were calculated according to the best fit model using CurveExpert Version 1.4 (Copyright 2013, Daniel G. Hyams) [21]. The absolute buffering capacity was also calculated in the same way and defined as the amount of sodium hydroxide needed to increase by one unit the initial pH value of the sample dispersion in normal saline. The same set of experiments was further performed using a vaginal fluid simulant (VFS) as the dispersion medium for the considered products. The VFS was prepared according to Owen and Katz [22] and contained sodium chloride (3.51 g), potassium hydroxide (1.40 g), calcium hydroxide (0.222 g), bovine albumin (0.018 g), lactic acid (2.00 g), glacial acetic acid (1.00 g), glycerin (0.16 g), urea (0.4 g), dextrose (5.0 g) and water (enough to complete one liter). The pH was adjusted to 4.2 with hydrochloric acid.

### 2.4. Osmolality Assessment

The osmolality of tested products was measured using a freezing point depression osmometer (Micro-Osmometer Type 15, Löser Messtechnik, Berlin, Germany) as described by others [13,15]. Whole samples were used except when osmolality values were above the maximum limit of the apparatus (2000 mOsm/kg). In these cases, products were diluted (1:1) with unionized water and the obtained osmolality values multiplied by the dilution factor. Preliminary experiments showed good agreement between osmolality values obtained before and after dilution of gels.

### 2.5. Cytotoxicity Studies

The cytotoxicity of tested products to HeLa cervical cells (ATCC, Manassas, VA, USA) was studied using a commercially available lactate dehydrogenase (LDH) colorimetric cytotoxicity assay kit (LDH Cytotoxicity Detection Kit, Takara Bio, Shiga, Japan) as previously described [23]. Briefly, HeLa cells (Passages 24–36) were maintained at 37 °C/5% CO_2_ in Dulbecco’s Modified Eagle Medium (Invitrogen, Carlsbad, CA, USA) supplemented with 10% fetal bovine serum (FBS; Invitrogen), 100 IU penicillin and 100 µg/mL streptomycin. Cytotoxicity testing was performed by seeding HeLa cells in 96-well plates (5000 cells/well) and incubating overnight. Next, cells were incubated for 24 h with different dilutions of products (1:5, 1:20 and 1:100, *w*/*v*, in medium without FBS), the plates centrifuged (1500 rpm, 10 min) and LDH levels evaluated in the cell supernatant according to the manufacturer’s protocol. Low (only medium) and high (2% Triton X-100) controls were included in order to calculate basal (0% toxicity) and maximum (100% toxicity) levels of LDH release, respectively. N-9 in aqueous solution was also included as a vaginal-relevant toxicity standard at a concentration commonly found in spermicidal products (2%, *w*/*v*) [24]. Further, half-maximal cytotoxic concentration (CC_50_) values were calculated by logistic regression using CurveExpert.

### 2.6. Statistical Analysis

All determinations were performed in triplicate unless otherwise stated. One-way ANOVA with Duncan’s *post hoc* test was carried out to assess differences between different products/conditions. All analyses were performed using SPSS 17.0 software (SPSS Inc., Chicago, IL, USA), and *p* ˂ 0.05 was accepted as denoting significance.

## 3. Results and Discussion

The maintenance of an acidic pH contributes to the normal vaginal physiology and microbiota, as well as to a balanced immune response. Vaginal products should present compatibility with vaginal pH and, ideally, maintain it or even help in its reestablishment (e.g., in cases of bacterial vaginitis or menopausal women) [25,26]. Results for pH of whole lubricants are presented in Table 1. Most of the tested products presented pH values in the acidic range. Values for Replens^®^ were in accordance to those previously reported (2.9–3.0), whereas in the case of K-Y^®^ Jelly, differences were notable (4.5–4.6) [15,16]. This last lubricant, curiously, has distinct compositions in different geographical regions, namely in Europe, the United States and South America, making comparison impossible. No data has been reported for the remaining lubricants. Among all products tested, only five were within the range values defined as normal for the vaginal milieu (3.5–4.5) [2]. Even so, most of the remaining deviated only marginally from this range, thus possibly limiting any putative deleterious effects. However, GelSea^®^ possesses a pH value that is inadequately high for vaginal administration. Even if the consequences of the administration of vaginal formulations presenting undesirable pH is not readily assessable, it is well known that increased vaginal pH is associated with the presence or favoring of bacterial vaginosis, trichomoniasis or mixed infections [27]. Outcomes of low pH are even less understood, but animal data suggest that values of three or less are unacceptable for human use [28].

**Table 1:** pH and osmolality values of tested products. Results are presented as the mean ± SD (*n* = 2–3).

Product	pH ^a^	Osmolality (mOsm/kg) ^b^
Fillergyn^®^	4.5 ± 0.1	991 ± 6
Geliofil^®^ Classic	3.8 ± 0.1	3,582 ± 11
GelSea^®^	5.7 ± 0.1	3,797 ± 16
Ginix^®^	5.0 ± 0.1	989 ± 9
Ginix^®^ Plus	5.0 ± 0.1	977 ± 8
Hyalo Gyn^®^	4.8 ± 0.1	1,336 ± 7
K-Y^®^ Jelly	3.5 ± 0.2	3,631 ± 13
Phyto Soya^®^	4.6 ± 0.1	1,226 ± 6
RepHresh^®^	3.4 ± 0.1	1,439 ± 6
Replens^®^	3.0 ± 0.1	1,177 ± 5
Velastisa^®^ Intim VG	3.7 ± 0.1	1,151 ± 7
Vidermina^®^	4.9 ± 0.1	3,707 ± 16
Universal Placebo	4.4 ± 0.1	298 ± 2

^a^ Desirable pH values are around normal vaginal values (3.5–4.5) [2]; ^b^ desirable and acceptable values are 380 and 1200 mOsm/kg, respectively, as recommended by the WHO [17].

**Figure 1:**
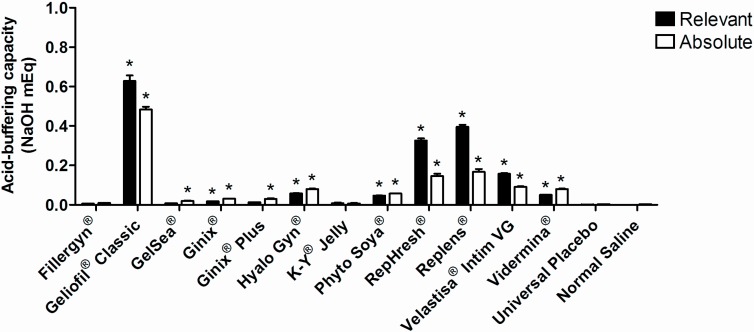
The relevant and absolute buffering capacity of tested products in normal saline. Individual columns and vertical bars represent mean and SD values, respectively (*n* = 3). (*) Denotes a significant difference (*p* ˂ 0.05) when comparing to the values of the relevant or absolute buffering capacity for plain normal saline.

Apart from being pH compatible, vaginal products should allow the maintaining of the vaginal acidic environment and oppose pH-raising events. Indeed, the use of acid-buffering gels has been proposed for the reestablishment of pH in cases of infection [29] or menopausal atrophy [30]. Buffering properties of selected products when dispersed in normal saline are presented in Figure 1. The majority of the gels presented significant relevant buffering capacity as compared to plain normal saline, except for Fillergyn^®^, GelSea^®^, Ginix^®^ Plus, K-Y^®^ Jelly and the Universal Placebo. Relevant buffering capacity was also low for Ginix^®^ and not different from Ginix^®^ Plus. The absolute buffering capacity was further calculated as being a more reliable means of measuring the buffering strength irrespective of the initial pH of the gel. For example, RepHresh^®^ and Replens^®^ seem to owe their higher relevant buffering capacity to starting low pH values (3.4 and 3.0, respectively). Overall, buffering capacity is probably related to the presence of acidic polymers in the composition of lubricants, even if the most acid-buffering product (Geliofil^®^ Classic) relies on the lactic acid/lactate buffer system (p*K*_a_ = 3.9) to maintain its pH balance. This low molecular weight acid may well be an ideal buffer system for vaginal formulations, since it is naturally produced *in vivo* by lactobacilli and bears the main responsibility for the acidic pH of the vagina [31,32].

**Figure 2:**
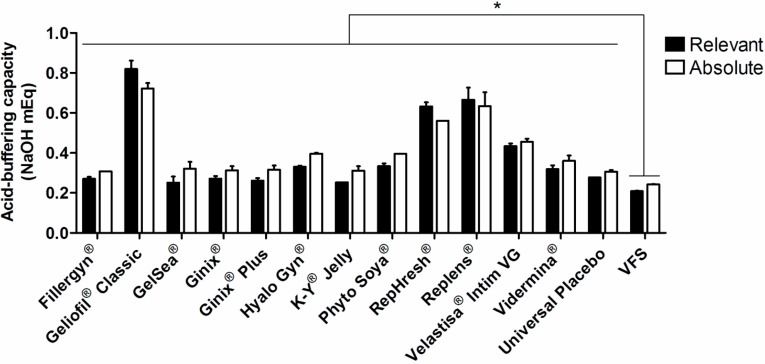
Relevant and absolute buffering capacity of tested products in vaginal fluid simulant (VFS). Individual columns and vertical bars represent mean and SD values, respectively (*n* = 2–3). (*) Denotes a significant difference (*p* ˂ 0.05) when comparing to values of relevant or absolute buffering capacity for plain VFS.

In order to better understand the real buffering ability of tested products on vaginal pH, the same set of experiments was performed using VFS as the dispersing medium. Results are presented in Figure 2. In general, the same trend was observed as for titration in normal saline, even if absolute differences between products were reduced. Geliofil^®^ Classic presented the highest relative and absolute buffer capacity, followed by Replens^®^ and RepHresh^®^. All products significantly increased the buffer ability of VFS, which may indicate a positive effect concerning pH maintenance. However, and even if VFS is recognized as having a low buffering ability [22], absolute differences from plain VFS were mild for most products, with a maximum four-fold increase in the case of Geliofil^®^ Classic. Moreover, this experimental setting did not evidence differences between relative and absolute buffer capacity values for the same product, which may suggest that more than the initial pH value of a formulation, the strength of the buffer system is of particular relevance.

Available pre-clinical and clinical data support that hyperosmolal vaginal products may be related to safety issues [13,16,33], as well as detrimental effects on sperm motility, viability and chromatin quality [34,35]. Results for osmolality (Table 1) showed that seven out of 12 tested commercially available gels did not comply with the minimum recommended standards from the WHO (˂1200 mOsm/kg) [17]. Four products presented values around three-fold higher than the maximum acceptable, while none fulfilled the ideal upper limit (380 mOsm/kg). These results are most probably related to the presence of high levels of glycerin and/or propylene glycol in the composition of all gels. Despite lacking quantitative information for most products, previous reports indicated that values of 1200 mOsm/kg for a HEC gel corresponded to an approximate content in glycerin of 10% (*w*/*w*) [13]. The osmolality value for Velastisa^®^ Intim VG (1151 mOsm/kg) was in agreement with its glycerin content (11.5%), as reported in the product insert. Indeed, the WHO recommends that glycerin and propylene glycol concentrations should not exceed 9.9% (*w*/*w*) and 8.3% (*w*/*w*), respectively. Previous osmolality results of 1491–2143 mOsm/kg and 2007–2510 mOsm/kg have been reported for Replens^®^ and K-Y^®^ Jelly, respectively, when acquired in the U.S. market [13,15,16]. Despite possible variations caused by the methodology used to evaluate this parameter, the value disparity observed in the present work, as well as among previous studies, seems to further reinforce regional differences in composition of the same brand name product. While changes in the qualitative composition of K-Y^®^ Jelly are evident, differences for Replens^®^ appear to be merely quantitative. Therefore, caution should be taken when translating literature data for lubricant products produced/acquired in different countries.

**Figure 3:**
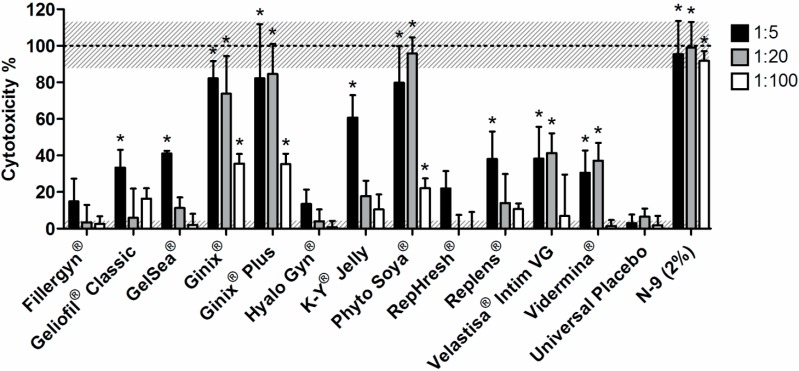
Cytotoxicity of tested products to the HeLa cell line after 24 h incubation at 1:5, 1:20 and 1:100 dilution ratios in medium, as assessed by the LDH assay. Individual columns and vertical bars represent mean and SD values, respectively (*n* = 3). Horizontal dotted lines at 0% and 100% correspond to low and high controls, respectively (associated gray shades stand for SD values). (*) Denotes a significant difference (*p* ˂ 0.05) when comparing to cells incubated with the Universal Placebo at the same dilution ratio.

Next, the cytotoxicity of lubricants was tested *in vitro*. Different *in vitro* cell toxicity assays have been useful for testing different semi-solid formulations intended for vaginal delivery. For example, Mahalingam and colleagues [36] tested the cytotoxicity of a microbicide gel to the VK2/E6E7 human vaginal cell line using a tetrazolium-based assay (MTS assay, Promega, Madison, WI, USA). An assay testing ATP content (CellTiter-Glo^®^, Promega) has also been used by others to determine the effect of different microbicide gels on the viability of the HEC-1-A human endometrial cell line [37,38]. Moreover, a previous study successfully used the LDH assay to test the toxicity of a microbicide gel to HEC-1-A cells [39]. The LDH assay is a standard in cell toxicity assessment and is based on the release of this cytoplasmatic enzyme upon damaging of the cell membrane [40]. Its levels can be readily assayed in the supernatant, providing an indicator of the cytotoxicity of tested products. In this study, the toxicity of lubricants to HeLa cervical cells was determined using the LDH assay, and the results are presented in Figure 3. Fillergyn^®^, Hyalo Gyn^®^ and RepHresh^®^ showed no significant toxicity at all tested dilutions, as compared to the Universal Placebo. Indeed, this last gel, which is considered a safety standard in vaginal microbicide drug delivery [18,19], presented a negligible increase in LDH levels as compared to the baseline. Moreover, and despite several design limitations, a recent clinical study on the use of Hyalofemme^®^ (commercial equivalent to Hyalo Gyn^®^) in postmenopausal women found that the product presented a generally high safety profile, without inducing changes to the vaginal microbiota [41]. Our *in vitro* results (Figure 3) seem to be in agreement with these *in vivo* observations.

Gel concentrations higher than 20% were not tested, as the viscosity of obtained dilutions was found impracticable for cytotoxicity testing. For Geliofil^®^ Classic, GelSea^®^, K-Y^®^ Jelly and Replens^®^, increased toxicity was only apparent at the highest concentration tested (Figure 3). In line with the finding for Replens^®^, Adriaens and Remon [13] demonstrated that this lubricant was able to induce mild irritation using a slug mucosal irritation assay. Further, Dezzutti *et al.* [16] showed nearly complete loss of HEC-1-A cell viability when Replens^®^ was incubated at 1:10 dilution, but no changes in viability, tissue architecture or HIV-1 susceptibility when tested in a polarized human ectocervical explant culture model. These last, somewhat divergent results seem to highlight the limitations of cell toxicity assessment methods, particularly related to their correlation with *ex vivo* or even *in vivo* situations, thus recommending caution when extrapolating data.

Ginix^®^, Ginix^®^ Plus and Phyto Soya^®^ were shown to possess the highest cytotoxicity with significant differences observed at all dilution levels, as compared to the Universal Placebo (Figure 3). The results were comparable to those of N-9 at 1:5 and 1:20 dilutions, but were significantly lower for 1:100 (*p* ˂ 0.05). Indeed, this last dilution corresponds to roughly 20-times the CC_50_ of N-9 to HeLa cells, as determined by our group using the LDH assay (unpublished data). Thus, the cytotoxicity of Ginix^®^, Ginix^®^ Plus and Phyto Soya^®^ seems to be still considerably lower than that of 2% N-9. Of particular interest, N-9 in the form of a gel has been previously shown to increase the risk of HIV-1 transmission in women undergoing a large-scale phase 2/3 clinical trial [42], an effect that has been correlated to the induction of mucosal inflammation and epithelial damaging [43].

**Figure 4:**
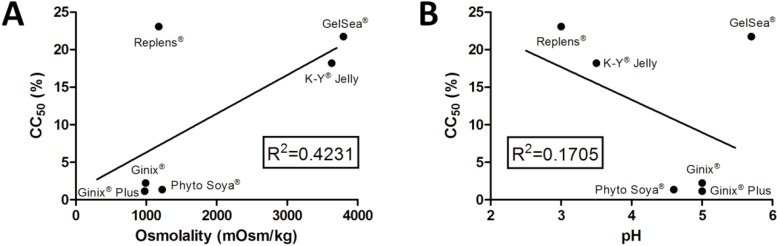
Correlation of cytotoxicity, expressed as half-maximal cytotoxic concentration (CC_50_) values, with the (**A**) osmolality or (**B**) pH of the selected products. Individual points are mean values, and the straight line corresponds to linear regression (*R^2^* values presented in the graphs).

Associations between cytotoxicity results and product characteristics are not easy, namely due to fairly different product composition (Table S1). The unknown quantitative description of lubricant ingredients further complicates this task. No correlation was found between CC_50_ values and osmolality, as shown in Figure 4. Lubricants presenting higher toxicity (Ginix^®^, Ginix^®^ Plus and Phyto Soya^®^) showed osmolality values (Table 1) around the maximum recommended by the WHO [17] and similar to those of products showing no toxicity (Fillergyn^®^, Hyalo Gyn^®^, RepHresh^®^). Further, Geliofil^®^ Classic and K-Y^®^ Jelly showed only toxicity at the 1:5 dilutions, despite having osmolality values above 3000 mOsm/kg. A previous study using a slug mucosal irritation test found a good correlation (second order polynomial regression) between increasing osmolality and higher irritation potential for both commercially available lubricants and glycerin-containing HEC gels [13]. Values of around 10% glycerin for HEC gels were found safe, corresponding to an osmolality value of 1493 mOsm/kg. Furthermore, Dezzutti and colleagues [16] tested an array of OTC lubricants and found a general trend for decreasing cell viability (HEC-1-A cells) with increasing osmolality. However, as apparent in the present study, outlier products were identified. Furthermore, none of the OTC lubricants tested in our work presented near *iso*-osmolality, which further limits the analysis.

Furthermore, there seemed to be a lack of correlation between CC_50_ values and pH (Figure 4). The only product possessing pH clearly out of the normal range for the vagina (GelSea^®^, pH = 5.7) had only limited toxicity, as compared to the Universal Placebo. Caution should be taken when interpreting pH values, since the LDH assay is performed at medium pH (7.2–7.4) and does not reflect the proton activity occurring in the vaginal milieu. Moreover, the buffer ability of the cell medium used was able to reduce pH differences between gels, namely in those presenting lower buffer capacity (Figure 2). Even for lubricants presenting higher buffer capacity, namely Geliofil^®^ Classic, RepHresh^®^ and Replens^®^, pH changes to media do not seem to reflect increased cytotoxicity.

The intrinsic toxicity of individual components of gels, in particular, may be more relevant in justifying toxicity results. Again, this analysis is limited due to a lack of information on the quantitative composition of the tested products. Half of the products tested contained one or more parabens. These preservatives have been shown to be more toxic than sorbic acid in *in vitro* assays relevant for vaginal drug delivery [44]. The results of this work were mixed regarding this comparison, but even so, the low toxicity of the Universal Placebo seems to recommend its use as safe. The products showing higher cytotoxicity (Ginix^®^, Ginix^®^ Plus and Phyto Soya^®^) have in their composition another preservative, phenoxyethanol. No studies concerning specific vaginal utilization are available, but a report on the safety of phenoxyethanol concluded that this ingredient is generally safe for cosmetic use, with only mild potential for causing mild skin or eye irritation upon topical administration of diluted solutions [45]. Still, Fillergyn^®^, which presented low cytotoxicity, possesses phenoxyethanol in its composition (Table S1). Again, the concentration may explain the toxicity outcomes.

## 4. Conclusions

Concerns have been raised about the safety of OTC vaginal lubricants. Lack of data, however, limits proper judgment on commercially available products, either by women or their assisting clinicians. The present study detailed the characteristics of different lubricants linked to vaginal safety. Most of the studied lubricants presented pH and/or osmolality values outside the ranges recommended by the WHO. Furthermore, cytotoxicity studies were able to identify safety concerns, even if the true impact of these findings requires further assessment. No definite correlation between gel pH or osmolality and cytotoxicity were found. Individual composition for tested products, as well as regional formulation variability, seems to be essential to undertake any specific cause-effect analysis. Further characterization is also deemed necessary in order to fully understand the potential hazard of the tested products, namely condom compatibility and safety to microbiota. In this last case, the ubiquitous presence of preservatives (parabens and others) in the composition of lubricants and the concentrations used may be of paramount importance. These and other excipients may interact differently with microbiota and trigger singular toxicity mechanisms, thus potentially leading to inconsistencies with cytotoxicity data obtained in the reported investigation for cells of epithelial origin. Thus, further specific toxicity testing using vaginal microbiota, namely *Lactobacilli* sp., is advisable. Overall, it seems highly recommended that regulatory agencies and manufacturers join efforts in the (re-)evaluation of commercial lubricants and, consequently, consider their eventual reformulation.

## Supplementary Files

Supplementary File 1
