# Supplementary material for: Characterization of Commercially Available Vaginal Lubricants: A Safety Perspective
Source: Pharmaceutics. 2014 Sep 22;6(3):530–42. doi: 10.3390/pharmaceutics6030530 (PMC4190534; doi:10.3390/pharmaceutics6030530)
Supplement: Supplementary File 1 [file pharmaceutics-06-00530-s001.doc]

**Supplementary Information**

**Table S1.** Detailed information of tested products.

| **Product** | **Manufacturer or Distributor** | **Purchased/Acquired from…** | **Ingredients** | **Indications/Claims** | **Condom  Compatibility ^a^** |
| --- | --- | --- | --- | --- | --- |
| Fillergyn® gel | BSDpharma, Lodi, Italy | Community pharmacy, Italy | Water, sorbitol, propylene glycol, glycerin, hydroxyethylcellulose, methylsulfonylmethane, benzyl alcohol, sodium hyaluronate, copolymer of methyl vinyl ether and maleic anhydride, Tea Tree oil (*Melaleuca alternifolia* essential oil), lactic acid, phenoxyethanol, methylparaben, ethylparaben, propylparaben, butylparaben disodium EDTA | Promotion of reparative processes of the atrophic vaginal mucosa during menopause | Yes (latex condoms) |
| Geliofil® Classic gel | Laboratoires Effik, Meudon-la-Forêt, France | Kindly provided by Effik, Portugal | Water, lactic acid, glycogen, propylene glycol, hypromellose, sodium lactate | Restoration of the natural balance of the vaginal flora | N.R. |
| GelSea® gel | LDPSA, Paris, France | Kindly provided by CP, Portugal | Water, glycerin, propylene glycol, hydroxyethylcellulose, carbomer,  *Spirulina maxima*, sodium hydroxide | Vaginal dryness, including during sex | Yes (latex condoms) |
| Ginix® gel | ISUS, Lisbon, Portugal | Kindly provided by ISUS, Portugal | Water, glycerin, sorbitol, phenoxyethanol, hydroxyethylcellulose, imidazolidinyl urea, carbomer, ethylhexylglycerin, xanthan gum, sodium hydroxide, sodium hyaluronate, *Arnica montana* extract,  *Calendula officinalis* extract, *Ribes nigrum* extract, *Salvia officinalis* extract, tocopherol | Vaginal dryness, including during sex | Yes |
| Ginix® Plus gel ^b^ | ISUS, Lisbon, Portugal | Kindly provided by ISUS, Portugal | Water, glycerin, sorbitol, hydrogenated lecithin, phenoxyethanol, hydroxyethylcellulose, imidazolidinyl urea, carbomer, ethylhexylglycerin, xanthan gum, sodium hydroxide, sodium hyaluronate, *Arnica montana* extract, *Calendula officinalis* extract, *Ribes nigrum* extract, *Salvia officinalis* extract, tocopherol | Vaginal dryness, including during sex | Yes |

**Table S1.** *Cont.*

| **Product** | **Manufacturer or Distributor** | **Purchased/Acquired from** | **Ingredients** | **Indications/Claims** | **Condom  Compatibility ^a^** |
| --- | --- | --- | --- | --- | --- |
| Hyalo Gyn® gel | Fidia Farmaceutici, Abano Terme, Italy | Community pharmacy, Italy | Water, Hydeal-D® (hyaluronic acid derivative), propylene glycol, carbomer 974P, methylparaben, propylparaben, sodium hydroxide | Hydration and lubrication to enhance the ease and comfort of intimate sexual activity | Yes (lubricated/ non-lubricated latex, lubricated polyurethane, and lubricated natural skin condoms) |
| K-Y® Jelly | Johnson & Johnson, Issy les Moulineaux, France | Community pharmacy, Portugal | Water, propylene glycol, sorbitol, hydroxyethylcellulose, benzoic acid, polysorbate 60, tocopheryl acetate | Easing of the discomfort of vaginal dryness during sex | Yes (latex condoms only) |
| Phyto Soya® gel | Arkopharma Laboratoires Pharmaceutiques, Carros, France | Community pharmacy, Italy | Water, glycerin, liquid paraffin, sodium hydroxide, carbomer, soya extract (*Glycine max*), phenoxyethanol, methylparaben, ethylparaben, butylparaben, propylparaben, isobutylparaben | Reduction of vaginal dryness and improvement of the sexual comfort, particularly  during menopause | N.A. |
| RepHresh® gel | Lil’ Drug Store Products, Cedar  Rapids, IA, USA | Community pharmacy, Italy | Water, glycerin, polycarbophil, carbomer homoploymer type B, sodium ethylparaben, sodium methylparaben, sodium propylparaben, sodium hydroxide | Elimination of vaginal odor and maintenance of healthy vaginal pH | Yes |
| Replens® gel | Lil’ Drug Store Products, Cedar  Rapids, IA, USA | Community pharmacy, Italy | Water, polycarbophil, mineral oil, glycerin, hydrogenated palm oil glyceride, carbomer homoploymer type B, sorbic acid, sodium hydroxide | Long-lasting vaginal moisturizer, for routine use and as a sexual lubricant | Yes |
| Velastisa® Intim VG moisturizer gel cream | Isdin, Barcelona, Spain | Kindly provided by Isdin, Portugal | Water, glycerin, polyglyceryl methacrylate, vaseline, liquid paraffin, hydrogenated palm oil glyceride, sodium hydroxide, carbomer, polyacrylic acid, methylparaben | Long-lasting vaginal moisturizer for managing vaginal dryness, including during sex | Yes |
| Vidermina® gel | Istituto Ganassini, Milano, Italy | Community pharmacy, Italy | Water, glycerin, propylene glycol, sorbitol, carbomer, sodium hyaluronate, hydrolyzed glycosaminoglycans, panthenol, *Chamomilla recutita* extract, arginine,  PEG-40 hydrogenated castor oil, imidazolidinyl urea, methylparaben, propylparaben, disodium EDTA | Genital lubricant (external  use recommended) | Yes |

^a^ According to manufacturer/distributor; ^b^ liposomal gel; N.A., not available; N.R., not recommended.

© 2014 by the authors; licensee MDPI, Basel, Switzerland. This article is an open access article distributed under the terms and conditions of the Creative Commons Attribution license (http://creativecommons.org/licenses/by/3.0/).
